# Is there an evidence for the role of multidisciplinary team in the management of active juvenile idiopathic arthritis?

**DOI:** 10.1186/1546-0096-12-S1-P177

**Published:** 2014-09-17

**Authors:** Sarka Fingerhutova, Melanie Saifridova, Marketa Vranova, Pavla Dolezalova, Stanislava Sebkova, Marek Bohm, Dana Nemcova, Jitka Obrsalova

**Affiliations:** 1 Paediatric Rheumatology Unit, General University Hospital in Prague, Prague, Czech Republic

## Introduction

Complex management of JIA comprises of drug treatment, physiotherapy/occupational therapy, education and counselling. It should be provided by a trained multidisciplinary team of physicians and allied health professionals (AHP). In the traditional model, nursing and physiotherapy staff competencies are limited by the leading role of physicians and support of an equally important role of AHP is not automatically provided. A case for the importance of AHP needs to be presented to the healthcare providers and hospital managers.

## Objectives

The project has 2 parts: 1.To develop a comprehensive system of therapeutic interventions provided by AHP to paediatric patients with JIA (a subject to this report). 2.To prospectively test performance of these interventions in a cohort of JIA patients with active disease requiring new drug treatment.

## Methods

Two trained rheumatology nurses and a physiotherapist contributed to the development of an AHP intervention plans as an add-on to the routine clinical care. Apart from the published literature the main resources included observations made by the team during their educational visits to the 2 European paediatric rheumatology services (Utrecht, NL, and Birmingham, UK). For the 2^nd^ part of the study patient inclusion criteria were: 1. Active JIA (at least 1 joint with active synovitis). 2. Newly diagnosed, untreated JIA or JIA relapse requiring medical intervention. Performance of interventions was tested by standardized quality of life assessments (CHAQ, parent/patient global assessment, SMILY-illness, JAMAR) along physician-derived disease activity measures (physician global, active joint count, ESR/CRP, JADAS71). Consecutive patients have been randomised into 3 groups according to the frequency of AHP interventions (3 or 6-monthly or no extra AHP intervention).

## Results

AHP intervention had 2 parts: an interview (about 30-45 min) and a practical session. Nurse interview covered 3 main areas: Introduction of the rheumatology team, patient history (schooling, relationships, hobbies and psychosocial aspects) and education (disease, its treatment, monitoring and parent/patient assessments). Where eligible, practical training in injection technique and drug safety/handling issues was provided. Physiotherapy (PT) interview covered history of pain, functional limitation, school PE, sporting, vocational issues and education on PT management. Practical input included full PT assessment, complex evaluation of physical function and establishment of the PT treatment plan. From November 2013 to May 2014 total of 41 consecutive patients were eligible from which one family refused participation. Polyarticular JIA was present in 19 children, oligoarthritis in 14, psoriatic, enthesitis-related or systemic JIA in 7 patients. There were 24 patients with the new diagnosis of JIA, 16 had JIA relapse. Median age at study entry was 6.5 years (3.7-10.1), prior disease duration was 1.0 year (0.2-3.7). Median active joint count at study entry was 3 (1-6.5), JADAS 71 was 9 (6-18). From 21 patients in whom the first F/U assessment was available, 10 received intraarticular triamcinolone-hexacetonide (in 2 cases with methotrexate, MTX), 6 received s.c. MTX and in 5 patients biologic therapy was added to MTX.

## Conclusion

With this study we aim to accumulate better evidence on the importance of the trained nursing and physiotherapy staff in the multidisciplinary team caring for rheumatology patients in the country where such an approach is not fully supported by the existing system. Evaluation of the performance of interventions provided by AHP is a subject of ongoing study.

## Disclosure of interest

S. Fingerhutova: None declared, M. Saifridova: None declared, M. Vranova: None declared, P. Dolezalova Grant / Research Support from: Grant IGA NT/14149-3 , S. Sebkova: None declared, M. Bohm: None declared, D. Nemcova: None declared, J. Obrsalova: None declared.

